# Multi-feature evaluation of financial contagion

**DOI:** 10.1007/s10100-021-00756-3

**Published:** 2021-06-18

**Authors:** Jarosław Duda, Henryk Gurgul, Robert Syrek

**Affiliations:** 1 Institute of Computer Science, Faculty of Mathematics and Computer Science, Jagiellonian University in Krakow, ul. Prof. S. Lojasiewicza 6, 30-348 Kraków, Poland; 2 Department of Applications of Mathematics in Economics, Faculty of Management, AGH University of Science and Technology, Gramatyka 10 St., 30-067 Kraków, Poland; 3 Institute of Economics, Finance and Management, Faculty of Management and Social Communication, Jagiellonian University in Krakow, ul. Prof. S. Lojasiewicza 4, 30-348 Kraków, Poland

**Keywords:** Contagion, Nonstationarity analysis, Conditional correlation, Hierarchical correlation reconstruction/HCR/, Principal component analysis, Feature extraction, G01, G11, G15

## Abstract

Financial contagion refers to the spread of market turmoils, for example from one country or index to another country or another index. It is standardly assessed by modelling the evolution of the correlation matrix, for example of returns, usually after removing univariate dynamics with the GARCH model. However, significant events like crises visible in one financial market are usually reflected in other financial markets/countries simultaneously in several dimensions, i.e., in general, entire distributions of returns in other markets are affected. These distributions are determined/described by their expected value, variance, skewness, kurtosis and other statistics that determine the shape of the distribution function of returns, which can be based on higher (mixed) moments. These descriptive statistics are not constant over time, and, moreover, they can interreact within the given market and among the markets over time. In this article we propose, and use for the daily values of five indexes (CAC40, DAX30, DJIA, FTSE250 and WIG20) over the time period 2006–2017, a new, simple and *computationally* inexpensive methodology to automatically extend contagion evaluation from the evolution of the correlation matrix to the evolution of multiple higher mixed moments as well. Specifically, the joint distribution of normalized variables for each pair of indexes is modeled as a polynomial with time evolving coefficients estimated using an exponential moving average. As we can obtain any arbitrary number of evolving mixed moments this way, its dimensionality reduction using PCA (principal component analysis) is also discussed, obtaining a lower number of dominating and relatively independent features, which can each be interpreted through a polynomial that describes the corresponding perturbation of joint distribution. We obtain features that describe the interrelations among stock markets in several dimensions and that provide information about the current stage of crisis and the strength of the contagion process.

## Introduction

It is clear that the activity of global investors has made stock markets throughout the world somewhat similar in various aspects. The fact that it is not easy to diversify one’s international portfolio results from this empirical observation. This is one of the most important motivations for studying the existence and strength of relationships between markets. The study of structure, channels of dependence, and relations between financial markets is essential for proper risk management and optimal portfolio allocation. This allows measures necessary for preventing a financial crisis or restricting its propagation between financial markets to be taken. In the last two decades these dependencies have been a topic in world financial and econometric literature that studies different types of short- and long-term linkages between stock markets, which in turn determine information flow between markets.

The processes on financial markets that have been observed, such as herding, trade linkages and financial linkages reflect the so-called contagion phenomenon. It can be noticed due to rapid spread from one market to another of declining prices and liquidity, increased volatility and increased correlation associated with shares from both markets. In the financial literature one cannot find a precise and unique definition of contagion on financial markets. The term contagion is well known from immunology. However, it has also been widely used in sociology and psychology. Recently it has found a new application in the analysis of financial crises and focused on different channels and aspects of widespread of them among companies and financial markets. The term was first used during the Asian financial crisis in 1997. However, this phenomenon occurred much earlier. The first global crisis was in 1929, i.e. the U.S. stock market crash, and is a very appealing example of the impact of contagion on the global economy. Research on contagion since 1997 has proven that, since roughly the England Latin America crisis of 1825, contagion has been visible in many crises which have taken place almost every decade.

In general, there are two main types of explanation of this, namely the fundamental reasons and investor behavior theories. The first group of contagion includes common shocks as outflow of capital from the emerging markets, significant changes in developed countries, a significant increase or decrease in commodity prices, a slowdown in global economic growth, and, mainly in developed countries, changes in trade interrelations after demand reduction. Strong regional interrelations support shock transmission from one country to its regional partners through e.g. capital flows and foreign investment.

The second group of contributions is concerned with contagion within the framework of investor behavior theories. The outbreak of a crisis on the domestic market can be the source of a liquidity problem for investors active on this market. To improve their liquidity the investors would probably try to sell their foreign assets. As a consequence, the prices of the foreign securities will decrease.

According to the theory of risk aversion of rational investors, turmoil on one emerging market may cause panic selling on other emerging markets, although there are no fundamental reasons. Overestimating the risk on an emerging market as a result of imperfect information and because it is asymmetrical causes foreign investors to leave emerging markets, which mostly is not justified by the results of evaluation of actual risk on these markets.

In the next section we will review the literature on contagion on stock markets with the focus on empirical results.

## Literature overview

In financial literature it is typically assumed that a significant rise in correlation or co-movement of the stock markets is a reflection of the contagion effect. King and Wadhwani (1990), Lee and Kim (1993) and Cha and Oh (2000) established on the basis of correlations that international stock markets interacted much more closely after the 1987 U.S. stock market crash and the Asian financial crisis in 1997 than some years before. According to these results, after these two crashes a continuous rise in co-movement between international stock markets was observed. Typically, the majority of international literature is oriented towards developed markets and concerned with the co-movement and interdependencies (comp. e.g. Hamao et al. 1990; Cappiello et al. 2006; Tilfani et al. 2019) between them.

A considerable part of literature tries to detect linkages between developed and emerging markets. These linkages were examined empirically in e.g. Chen et al. (2002), Kim et al. (2005), Syllignakis and Kouretas (2011), Ho and Huang (2014). Only a small part of the literature is concerned with the dependencies between stock markets in Europe. Moreover, the conclusions from these studies are not unique.

In the long term, the dependencies between European markets were the subject of contributions by e.g. Voronkova (2004), Černý and Koblas (2005), Égert and Kočenda (2007), Syriopoulos (2007), Czapkiewicz and Wójtowicz (2017). Most of these contributions detect interrelations between the daily returns of developed and emerging European stock markets operating in CEE countries, using cointegration and fractional cointegration.

In the short term more typical results known from the early literature on the topic can be observed. Hanousek et al. (2009) discovered an essential spillover impact on the stock markets of CEE countries. In their next contribution Égert and Kočenda (2011) found time dependent correlations between the intraday returns of indexes BUX, PX50, and WIG20.

An abnormal strong or weak correlation between stock markets is important with respect to portfolio diversification. In the literature, one of the most important factors observed on financial markets is the dynamics of dependence between markets during quiet and turbulent time periods. Insignificant changes in correlation during a calm period suggest that diversification should be undertaken. The rising correlation between markets after the beginning of a crisis implies that a decline on one stock market may be accompanied by a decline on the others. This is a result of the frequently observed contagion effect between stock markets. It is not possible to create a well-diversified portfolio without checking for possible contagion.

Before the empirical investigation, we will define the notion of “contagion” more precisely. In the financial literature there is no unique and widely accepted formal definition of contagion on financial markets. Therefore, there are different measures of contagion that underly different specific definitions of this phenomenon. The different approaches to contagion are reflected in different measures used to assess its strength and importance.

We will point out general relations between interdependence and contagion on financial markets. In the literature, the contagion is often understood just as propagation of shocks, but this is not fully justified. It is true that usually correlations of returns react to unexpected shock or event (Murg et al. 2016; Gurgul and Majdosz 2007; Gurgul and Wójtowicz 2014).

The contributors Castellano and Scaccia (2014) suggest that fluctuations in CDS indexes can signal the occurrence of future turmoils in the stock market. Such turmoils can be initial point of contagion process.

According to the literature (see, for example, Forbes and Rigobon 2002) contagion can be identified if dependencies between markets are stronger during turbulent times than in quiet times. Forbes and Rigobon (2002) introduced a useful distinction between non-crisis-contingent theories and crisis-contingent theories. They distinguish the different channels of shocks in the case of these two groups of theories. Theories of the first kind are those that explain the reason for the change in transmission channels and mechanisms during a crisis. In this way the authors try to explain why cross-market linkages increase after a shock. The second group of theories assume that channels and transmission mechanisms do not change during a crisis. Thus, the linkages detected are the same after a shock (Nieh et al. 2011).

We stress that the contagion effect does not occur when two markets are essentially correlated during turbulent and quiet periods. It can be detected in a situation where markets are more strongly connected in turbulent times than during quiet periods. Therefore, the contagion effect between two markets can be observed when a significant increase in correlation takes place during a turbulent period.

The World Bank has formulated three definitions of contagion, namely a broad definition, a restrictive one, and a very restrictive one (Kao et al. 2019). The broad definition of contagion is related to the cross-country transmission of shocks. In this case general cross-country spillover effects can be detected. This definition emphasizes that contagion does not need to be related to crises. The restrictive definition of contagion assumes the transmission of shocks to other countries or a cross-country correlation, which cannot be explained by fundamental interdependence between countries and due to common shocks. This definition is based on excess co-movement, usually explained in finance literature by herding behavior. The very restrictive definition of contagion assumes the detection of contagion when cross-country correlations increase during “crisis times” in comparison to correlations during “quiet times” (Nieh et al. 2011).

According to crisis contagion theory a co-movement, or a common trend between different markets, implies that a shock in one market would affect another market. Dornbusch et al. (2000) understood contagion as a significant increase in cross-market interdependencies after a shock affected an individual country or market, given by the size to which stock prices or financial flows moved together over the markets in comparison to co-movement in calm times.

The focus of a number of other studies that consider contagion is based on data concerning the financial crisis periods (see e.g. Candelon et al. 2005; Corsetti et al. 2005; Dimpfl and Peter 2014; Nam et al. 2008 or Yilmaz 2010, among others).

In the more recent study in this group, conducted by Dimpfl and Peter (2014), only partial evidence for the prevailing position of the US market before the crisis was found. From this study it follows that interrelations between the US and the German and the US and the UK markets became more balanced during and after the financial crisis. However, throughout the entire period under investigation a significant impact of the US market on the French market can be seen. In the opinion of these authors, information processing is nowadays conducted simultaneously at the European and the US stock exchanges on the basis of the results for intraday data. Thus, information flows between the different markets are of similar strengths. The finding that the relationship is not stable over time with respect to changing co-movement and information transmission between stock markets is in line with expectations. The authors stress that information, such as the collapse of Lehman Brothers, enhanced and sped up information flow and as a consequence had a significant effect on all markets. This proved that important pieces of information can cause feedback effects and information cascades. This may be the source of significant cases of market turmoil. To summarize, Dimpfl and Peter’s paper (2014) is in favor of contagion and spillovers between financial markets, especially in hectic times.

The most frequently used research method when assessing contagion effects is causality with respect to mean returns. This is because the interdependence between stock returns is usually proved in research projects. Only some of them, such as Ho and Huang (2014), refer to causality in variance. A paper by Abdennadher and Hellara (2018) analyzes the interconnections of stock market volatilities on capital markets. The authors try to assess the effects of the Global Financial Crisis (GFC) on the dynamics of these dependencies over the period concerned.

Jung and Maderitsch (2014) conducted a research project on contagion in volatility on the basis of the intraday data of the Hong Kong, Europe and United States stock markets over the period from 2000 to 2011. The authors computed a time series of realized volatility for these markets and, on the basis of a Heterogeneous Autoregressive Distributed Lag Model, conducted the respective calculations. They detected time-dynamics and structural breaks in volatility spillovers. In their conclusions for the time period of the 2007 financial crisis, they stressed that their results are in line with the notion of contagion. The study suggested essential and unexpected increases in the cross-market synchronization of the time series volatilities. They detected breaks in means and conditional heteroskedasticity in the realized time series of volatilities. In addition, they established that conditional heteroscedasticity seems to be the main reason for breaks in volatility spillovers. After the impact of the realized volatilities had been removed, the authors did not detect contagion anymore.

Most empirical studies concerning contagion effects are based on data from periods around financial crises. The simplest method of contagion detection is to compare correlations between markets before and after a crisis. One of the methods of comparison can be conducted using conditional correlation (CC) models. On the basis of Constant Conditional Correlation (CCC) and Smooth Transition Conditional Correlation (STCC) models, Savva and Aslanidis (2010) demonstrated that the stock markets in Poland, Hungary and the Czech Republic reflected stronger correlations with the Euro area than with small CEE markets.

Using the Dynamic Conditional Correlation (DCC) GARCH models, Syllignakis and Kouretas (2011) proved that the 2007–2009 global financial crisis essentially influenced the conditional correlation between the leading developed markets (Germany and US) and emerging CEE markets. In addition, Baruník and Vácha (2013) investigated contagion effects between CEE markets by mean of wavelets. They detected that the contagion effect is only between the German and Czech stock markets.

Investigating contagion effects on the basis of a comparison of correlations before and after a crisis has a significant drawback. It is connected with significance tests for the shift in correlation during these two periods. The source of this problem originates in different properties of the financial time series in the quiet and turbulent phases of financial markets. Therefore, Durante and Jaworski (2010), Durante et al. (2013), Durante et al. (2014) and Durante and Foscolo (2013) developed a new method for analyzing changes in stock market co-movements. They introduced the notion of spatial contagion with the framework of copula methodology. By comparing the correlation for extremely low returns (in the left tail of the returns distribution) with the correlation around the median, they replaced correlation comparison before and after a crisis. This definition of contagion is more general. It does not depend on the example of a particular crisis. It takes into account all cases of extreme losses on the markets under investigation. These results do not contradict the conclusions of Longin and Solnik (2001). On the basis of extreme value theory they found a higher correlation in the case of large negative returns.

In a paper by Brzeszczynski and Ibrahim (2019) a trading strategy is suggested based on fuzzy logic rules. The aim of this strategy is to quantify the size or direction of signals reflected in the inter-regional transmission effects in returns, and to determine how this impacts the performance of domestic trades in the six main stock markets in the U.S., Europe and Australasia. The channels of foreign information transmission (FIT) are modelled by the FIT model developed by Ibrahim and Brzeszczynski (2009). The authors quantified the domestic momentum. Its magnitude was measured using the Relative Strength Index. They constructed a trading system based on both foreign and domestic information signals. They used filters in order to measure the size and direction of the trading signals and determine the incremental impact on the economic performance of the suggested investment system. The results of Ibragim and Brzeszczynski (2014), based on FIT models, demonstrated that overnight international information is a better source of information than the previous day's domestic information. In the opinion of these authors, better modelling of the time variation in the impact of this overnight information is beneficial to investors.

In a more recent contribution Massad and Andersen (2018) introduce and explain methods to detect the dynamics of three different channels, in which synchronizing human decision-making can have serious consequences for stock markets. It could lead to crisis periods and contagion in financial markets. The first channel can be noticed when stock market indices try to synchronize in frequency. The authors call this kind of market behavior “integrate-and-fire dynamics”. It takes place due to “change blindness”, which is a property of human decision-making. This is reflected in the tendency to ignore small changes. Most people react when a large change takes place. The second channel takes place due to feedback mechanisms between market behavior and the application of selected trading strategies. The third channel can be detected due to the effects of communication and its effect on human decision-making. Massad and Andersen (2018) introduced a model in which financial market behavior has an influence on the decision-making process. This is possible due to communication among people. And, conversely, the sentiment caused by communication has an effect on the financial market situation. The authors cited methodologies useful in this kind of investigation. They included communication models of human decision-making, agent-based modeling and models of integrate-and-fire oscillators. Much attention to human decision-making observed as insider trading with respect to experimental trading patterns, manipulation, and profitability is paid in contribution by Hornung et al. (2015).

In the literature authors try to measure contagion effects using different tools, as well as using statistical tests. Fry-McKibbin et al. (2019) derived the new joint tests which were used in research on a broad spectrum of contagion problems on Euro-zone equity markets. The research was based on data from three financial crises: the subprime crisis of 2007–08, the global financial crisis (GFC) of 2008–09 and the European debt crisis of 2010–14. The tests known in the literature focus on single channels of transmission of crises. The authors checked the finite sample characteristics of the new tests. Moreover, they compared them with existing tests of contagion that focus on a single channel. They demonstrated that contagion can be detected when higher order moment channels are computed. This took the place during the GFC and the European debt crisis. For the sample under investigation in some cases the contagion found using new tests could not be detected via traditional tests based on correlations.

Linear correlation is not the only tool for identifying contagion phenomena, as there are also other statistical dependencies we can describe. This is possible using e.g. copulas. In their most recent publication Huynh et al. (2020) analyzed the contagion risk for the stock returns of listed commercial banks using non-parametric and copula tools. The results confirmed that the risk of these banks may be transmitted to other banks due to stock returns. The latter can be noticed in their price information. This paper also suggested a contagion risk. Another observation was a strong correlation with respect to the structure of the stock returns of these banks.

The interrelations between financial markets are considered in different aspects, i.e. the geography of market and political integration, international portfolio diversification, contagion and the predictive power of information transmitted from various financial markets. In our study we are concerned with the impact of selected important events on one financial market on other financial markets. Geographically we restricted our attention to stock markets from the transatlantic region of economic and political integrations.

As emphasized in the literature review, contagion is frequently reflected simultaneously in several dimensions, e.g. in average returns, volatility, skewness or kurtosis. These parameters defined by moments determine the shape of the distribution function of returns. The phenomenon of contagion makes distribution curves (also profit/losses profiles) on markets affected by contagion more similar to those of the returns on a particular financial market which has experienced shock(s) for the first time.

Our approach combines contagion considered in the literature separately via returns or volatility in unified, complex manner reflecting simultaneously all possible symptoms of contagion inclusive changes in returns and volatility.

In our study we propose a new approach not known in the literature for estimating the evolution of mixed moments, and apply it to studying contagion between stock markets represented by the following indexes: CAC40, DAX30, DJIA, FTSE250 and WIG20. Regarding the selection of indexes, we simply choose, to some extent in line with the ideas of Dimpfl and Peter (2014), and Yarovaya et al. (2020), one of the main indexes of the countries under investigation. These indexes have different capitalization sizes, but are among the most important in a given country. The WIG20 and FTSE250 indexes have a much smaller capitalization than the CAC40 and DAX30, and these in turn have a much lower capitalization than the DJIA. The empirical facts (e.g. strong reaction of different stock markets to U.S. macroeconomic announcements) prompt us to suppose the US stock market to be the most influential and least sensitive capital market in relation to other stock markets. The least stable market and most sensitive seems to be the Warsaw Stock Exchange, which represents emerging markets. The remaining European indexes represent large, developed economies with rather stable stock markets, but not as influential as US stock markets. The choice of these indexes of different sizes allows us to test the explanatory power of the methods used and to establish to what extent the complex characteristics of the stock markets can be transmitted pairwise via moments from one market to another regardless of the capitalization levels of its counterparts.

In the next section of this paper we present two types of methodology used in this study in order to detect and quantitatively describe contagion effects: a traditional methodology based on GARCH and DCC—copula modelling evolution of correlation matrix, and a new HCR (hierarchical correlation reconstruction) approach, also with PCA-based interpretable dimensionality reduction. The method we propose is somewhat related to that described by Fry-McKibbin et al. (2019) since it also takes different channels of contagion into account. However, the new approach is superior because it reflects the transmission of contagion through all possible channels simultaneously.

The remaining part of the paper is organized in sections and subsections. The traditional methodology used in this study is outlined in Sect. 3.1. The new approach is presented in detail in Sect. 3.2. Data is presented in Sect. 4. Section 5 makes reference to the methods in Sects. 3.1 and 3.2 and summarizes empirical results based on both approaches, and also on a number of plots. The last section provides a conclusion.

## Methodology

This section overviews the methodology and models used in the empirical analysis. Firstly, there is a description of univariate and multivariate GARCH models along with copulas, or the proposed HCR, to model the evolution of the higher mixed moments as well.

### Conditional correlation models and copulas

The main idea of conditional correlation models is to decompose the conditional covariance matrix into conditional standard deviations and correlations. In this way univariate and multivariate dynamics are separated. Bollerslev (1990) introduced a model in which the conditional covariance matrix is the product of conditional standard deviations matrices and the constant correlation matrix. Engle (2002) and Tse and Tsui (2002) introduced models in which the correlation matrix is time-varying. In both approaches, univariate dynamics is modelled with GARCH type models with an assumption of multivariate normality. Engle’s model has been the source of many generalizations, including asymmetric dynamics and non-elliptical multivariate distributions.

Abe Sklar (1959) introduced copula functions which are multivariate distributions with uniform margins. He proved that any multivariate distribution can be expressed in terms of univariate distributions and the copula. In this way the copula describes the dependence structure among random variables. Patton (2006) in his paper proved that Sklar theorem is valid for the conditional case, which leads to dynamic models based on copulas. An example of interesting and useful applications of copulas in finance is reported in contribution by Pflug and Pichler (2018).

In this paper we use a combination of Engle’s dynamic conditional correlation model (2002) and $t$ copula^1^ (computations are made using the R package rmgarch by Alexios Ghalanos). Let $r_{t}  = \left( {r_{{1t}} , \ldots r_{{nt}} } \right)'$ be the vector of returns of indexes with joint distribution $F$ such that:

1 $$ F\left( {r_{t} |\mu _{t} ,h_{t} } \right) = C\left( {F_{1} \left( {r_{{1t}} |\mu _{{1t}} ,h_{{1t}} } \right), \ldots ,F_{n} \left( {r_{{nt}} |\mu _{{nt}} ,h_{{nt}} } \right)} \right) $$

where $C$ is the copula function and $F_{i}$ are distributon functions. The terms $\mu _{{it}}$ and $~h_{{it}}$ refer to conditional mean and conditional variance, respectively. We use standard GARCH(1,1) specification with skew $t$-distribution $F_{i}$ of Fernandez and Steel (1998) with skew parameter $\xi _{i}$ and shape parameter $\nu _{i}$. The univariate models are used to obtain uniform margins: $u_{{it}}  = F_{{it}} (r_{{it}} |\mu _{{it}} ,h_{{it}} ,\xi _{i} ,\nu _{i} )$ for $i = 1, \ldots ,n$. The dependence structure is described using Student’s *t* copula with time-varying correlation $\user2{R}_{\user2{t}}$ and constant parameter $\upsilon$. Given $f_{t} \left( { \cdot {\text{|}}\user2{R}_{\user2{t}} ,\upsilon } \right),\,{\text{the}}~$ density of multivariate $t$ distribution and $f_{i} \left( { \cdot {\text{|}}\upsilon } \right),~$ the density of univariate margins of the multivariate $t$ distribution, the density of $t$ copula is given by:

2 $$ c_{t} \left( {u_{{1t}} , \ldots ,u_{{nt}} {\text{|}}\user2{R}_{\user2{t}} ,\upsilon } \right) = \frac{{f_{t} \left( {t_{\upsilon }^{{ - 1}} \left( {u_{{1t}} |\upsilon } \right), \ldots ,t_{\upsilon }^{{ - 1}} \left( {u_{{nt}} |\upsilon } \right){\text{|}}\user2{R}_{\user2{t}} ,\upsilon } \right)}}{{\mathop \prod \nolimits_{{i = 1}}^{n} f_{i} (t_{\upsilon }^{{ - 1}} \left( {u_{{it}} |\upsilon } \right)|\upsilon )}}. $$

where $t_{\upsilon }^{{ - 1}} \left( { \cdot |\upsilon } \right)$ is a quantile transformation of the uniform margins in respect to the common shape parameter of multivariate density. The dynamics of the correlation matrix can be described by DCC(1,1) Engle’s model (Engle 2002) and we adopt these settings. This new alternative methodology will be presented in the following subsection.

### Hierarchical correlation reconstruction (HCR)

It is convenient to estimate probability density as a linear combination from orthonormal basis (e.g. Chen 1979; Mathai and Haubold 2008), usually considered for univariate variables. HCR methodology, discussed e.g. in Duda (2018), Duda et al. (2020), Duda and Bhatta (2020), is a practical approach to apply it to multivariate case thanks to first normalizing variables to nearly uniform (marginal) distributions, as in copula theory.

Such a combination of polynomial density estimation with a copula approach allows one to focus on a detailed description of statistical dependencies between such $d$ variables as perturbation from $\rho  = 1$ uniform distribution on $\left[ {0,1} \right]^{d}$. This description can be constructed in a hierarchical way: of statistical dependencies between a growing number of variables: starting with correction to marginal distributions e.g. due to non-stationarity, then adding pairwise dependencies, then triple-wise etc.

The estimated coefficients—of orthonormal polynomials for normalized variables, have similar interpretations as cumulants, additionally allowing a model of joint distribution to be reconstructed. Here we will estimate these coefficients using EMA (exponential moving average) to model non-stationarity through the evolution of these cumulant-like coefficients.

As in copula theory, we will work on variables normalized to nearly uniform on $\left[ {0,1} \right]$ marginal distributions (with univariate dynamics removed by transforming through CDF with parameters from GARCH model as for DCC above). The values of these normalized variables can be imagined as quantiles of the original variable, e.g. $x = 1/2$ corresponds to the median.

Then, instead of usually single-parameter family of densities for copulas, we consider modelling of joint distribution in $$\left[ {0,1} \right]^{d}$$ for such normalized variables as a linear combination, e.g. in the product basis of orthonormal polynomials—for example for $$d = 2$$ variables (being in focus of this article) and considering up to $$m$$-th moments (e.g. up to kurtosis for $$m = 4)$$:

3 $$ \rho \left( {x,y} \right) = \mathop \sum \limits_{{j,k = 0}}^{m} a_{{jk}} ~f_{j} \left( x \right)~f_{k} \left( y \right)~ $$

where $\left\{ {f_{j} } \right\}$ is a orthonormal basis on $\left[ {0,1} \right]$: satisfying $\mathop \smallint \nolimits_{0}^{1} f_{j} \left( x \right)~f_{k} \left( x \right)~dx = \delta _{{jk}}$. We will use (Legendre) orthogonal polynomials for [0,1] range here up to $m = 4$:

$$ f_{0}  = 1 ,f_{1}  = \sqrt 3 \left( {2x - 1} \right),f_{2}  = \sqrt 5 \left( {6x^{2}  - 6x + 1} \right),f_{3}  = \sqrt 7 \left( {20x^{3}  - 30x^{2}  + 12x - 1} \right),~~f_{4}  = 3\left( {70x^{4}  - 140x^{3}  + 90x^{2}  - 20x + 1} \right). $$

Such model of density model as a polynomial can generally drop below zero $\left( {\rho \left( {x,y} \right) < 0} \right)$, which might require repairation like using e.g. $\bar{\rho } = \max \left( {\rho ,\epsilon} \right)/N$ density model instead for some $\epsilon > 0$ and normalization $N$ to integrate to 1, discussed e.g. in Duda et al. (2020).

However, while this joint density model can help e.g. with portfolio optimization, risk management or Monte Carlo simulations, in this article we are only interested in the behavior of these $\left\{ {a_{{jk}} } \right\}$ coefficients—as features which can be used for further analysis, e.g. detection of significant events. They have a similar interpretation as mixed moments of these variables, intuitively allowing for the hierarchical decomposition of statistical dependencies into a potentially infinite sequence of mixed moments, obtaining highly parametrized copula-like models, chosen e.g. on the basis of cross-validation.

As $f_{0}  = 1,~$ coefficient $a_{{00}}  = 1$ corresponds to normalization to integrate to 1. Then $a_{{10}} ,a_{{20}} ,a_{{30}} ,a_{{40}}$ describe the marginal distribution of the first variable (they should be zero if perfectly normalized to uniform distribution)—they have similar interpretation as the correspondingly expected value, variance, skewness and kurtosis. Analogously $a_{{01}} ,a_{{02}} ,a_{{03}} ,a_{{04}}$ for the second variable.

Further, $a_{{jk}}$ with $j,k \ge 1~$ intuitively describe the dependencies between $j$-th moment of the first variable and $k$-th moment of the second, starting with $a_{{11}}$ similar to the correlation coefficient, $a_{{22}}$ to dependence of variances.

While these were symmetric (do not depend on the order of variables), we also have asymmetric mixed moments that allow the directional statistical dependencies to be evaluated, starting with $a_{{12}}$, which describes the growth in variance of the second variable with a change of value in the first.

We can analogously work with 3 or more variables, e.g. $a_{{110}}$ describes the correlation between the 1st and 2nd out of 3 variables, $a_{{011}}$ between the 2nd and 3rd. Then positive $a_{{111}}$ says that with the 1st variable above the median, the 2nd and 3rd are positively correlated, and negatively for the 1st variable below the median. Thus for $n$ variables, to consider up to $m$-th mixed moments of all $k \le n$ subsets of variables, we would need $$( {\begin{array}{*{20}c}    n  \\    k  \\   \end{array} } )~m^{k}$$ coefficients—which in practice requires a basis of mixed moments to be optimized, as discussed e.g. in Duda et al. (2020).

While $m = \infty$ infinite series would in theory allow nearly any joint distribution to be represented, besides the infinite computational cost, such an estimation would require an infinite dataset. In practice we can optimize the basis selection on the basis of e.g. cross-validation. Considering those up to kurtosis $\left( {m = 4} \right)$ often turns out to be a good compromise—for simplicity we will focus on it here.

The use of an orthonormal basis allows for very simple and inexpensive MSE (mean-squared error) estimation, which leads to a formula for such coefficients as just average of corresponding function over the dataset:

4 $$ a_{{jk}}  = \frac{1}{T}\mathop \sum \limits_{{t = 1}}^{T} f_{j} \left( {x_{t} } \right)~f_{k} \left( {y_{t} } \right). $$

#### Evolving density for the nonstationary case

Especially for the contagion analysis, we would like to evaluate the evolution of joint distribution—described by such $a_{{jk}} \left( t \right)$ coefficients which now can evolve in time $t$:

5 $$ \rho _{t} \left( {x,y} \right) = \mathop \sum \limits_{{j,k = 0}}^{m} a_{{jk}} \left( t \right)~f_{j} \left( x \right)~f_{k} \left( y \right).~ $$

For their MSE estimation, a simple approach is to replace the average in estimation with the exponential moving average—locally increasing the weights of recent values:

6 $$ a_{{jk}} \left( {t + 1} \right) = \eta ~a_{{jk}} \left( t \right) + \left( {1 - \eta } \right)~f_{j} \left( {x_{t} } \right)~f_{k} \left( {y_{t} } \right). $$

Choosing above $\left( {\eta ,1 - \eta } \right)$ coefficients as summing to 1, weight of contribution $\Delta t$ time ago is $\left( {1 - \eta } \right)\eta ^{{\Delta t - 1}}$, which asymptotically sums to $\mathop \sum \nolimits_{{\Delta t = 1}}^{\infty } \left( {1 - \eta } \right)\eta ^{{\Delta t - 1}}  = 1$ as required for averaging.

The choice of $\eta$ forgetting rate is again a difficult one, fortunately evaluation is often nearly unchanged for a wide range of this coefficient like $\eta  \in \left( {0.97,~0.99} \right)$. It can be optimized so it is the same for all coefficients e.g. to maximize log-likelihood (e.g. in Duda and Bhatta 2020), can alternatively be optimized individually for each coeffcient e.g. with MSE. Here we could additionally optimize it separately for each pair of indexes. For simplicity and universal intepretation, as a compromise we use fixed $\eta  = 0.98$ in this analysis, in the middle of the safe range.

We also need to choose the initial values in $$t = 1$$, for simplicity chosen as $$a_{{jk}} \left( 1 \right) = 0$$ here (beside normalization $$a_{{00}}  = 1)$$, so their evolution starts with 0 and the initial period is less credible—to weaken this inaccuracy, values from 2006 were additionally used to initiate the model. This could be improved e.g. by using a two-sided moving weighted average instead, but it would require knowledge of future values—it can be used for analyzing historical data, while the presented approach can be implemented online, using only past data for evaluation.

#### Feature extraction with PCA (principal component analysis)

While $\left( {a_{{jk}} } \right)_{{j,k = 0..m}} ~$ can be interpreted as mixed moments, $a_{{jk}} \left( t \right)$ as their time evolution, e.g. for $m = 4$: considering up to kurtosis, we obtain $\left( {4 + 1} \right)^{2}  = 25$ of them ($4^{2}  = 16$ if neglecting marginals)—it would be valuable to extract just a few dominating and relatively independent features from them. We can use feature extraction techniques for this purpose, such as PCA (principal component analysis), previously discussed for multi-feature autocorrelation HCR analysis in Duda and Bhatta (2020).

Treating all $a_{{jk}} \left( t \right)$ as matrix e.g. $T \times 25$ here (for $m = 4$ considered moments up to kurtosis), for PCA we find its $25 \times 25$ covariance matrix $C$, and calculate its eigenvalues and eigenvectors (forming an orthonormal basis): $P = \left\{ {\left( {\lambda ,v} \right):Cv = \lambda v} \right\}$.

Then we can translate this density evolution into a new orthonormal basis:

7 $$ \rho _{t} \left( {x,y} \right) = \mathop \sum \limits_{{\left( {\lambda ,v} \right) \in P}} a_{v} \left( t \right)~f_{v} \left( {x,y} \right) $$

8 $$ {\text{for}}\quad f_{v} \left( {x,y} \right) = \mathop \sum \limits_{{j,k = 0}}^{m} v_{{jk}} ~f_{j} \left( x \right)f_{k} \left( y \right),\quad a_{v} \left( t \right) = \mathop \sum \limits_{{j,k = 0}}^{m} v_{{jk}} ~a_{{jk}} \left( t \right). $$

Thus $\lambda$ corresponds to variance of $\left\{ {a_{v} \left( t \right):t = 1, \ldots ,T} \right\}$. For feature extraction it is natural to choose a few $\left( {\lambda ,v} \right)$ pairs corresponding to the highest eigenvalues—which have the largest variances and are relatively independent due to orthogonality. Density $f_{v} \left( {x,y} \right)$ provides an interpretation for each such $a_{v} \left( t \right)$ evolving feature: as the direction of the perturbation of joint density described by a given feature.

## Data

Our computations used the data of several indexes and refer to selected events important for stock markets. The main goal of our study is to prove the contagion level around these events empirically by means of two methods. The first of them is based on the DCC model, and the second one is a new, comprehensive method developed by the authors.

### Descriptive statistics

Our dataset contains daily prices of five indexes covering subperiods 2006–2017 and 2018–2021: CAC40 (CAC), DAX30 (DAX), DJIA (DJI), FTSE250 (FTSE) and WIG20 (WIG). On the basis of daily prices at close we compute daily logarithmic returns in percentage. Table 1 contains the main descriptive statistics along with the results of autocorrelation (Ljung-Box test) and normality (Jarque–Bera test) testing.

**Table 1:** Descriptive statistics and testing results (*p* values)

Statistic	2006–2017					2018–2021				
	CAC	DAX	DJI	FTSE	WIG	CAC	DAX	DJI	FTSE	WIG
Mean	0.00	0.03	0.03	0.03	−0.01	0.02	0.02	0.04	0.01	−0.03
std. dev	1.48	1.43	1.16	1.19	1.51	1.39	1.43	1.53	1.28	1.52
Kurtosis	8.96	9.08	13.93	7.46	6.96	18.73	17.94	21.28	14.73	14.80
Skewness	−0.05	−0.09	−0.17	−0.44	−0.36	−1.34	−0.98	−1.02	−0.74	−1.12
L-B	0.01	0.50	0.00	0.00	0.00	0.00	0.00	0.00	0.00	0.01
J-B	0.00	0.00	0.00	0.00	0.00	0.00	0.00	0.00	0.00	0.00

Source: Authors calculation

In all cases we observe high kurtosis values and negative skewness. A departure from the norm is confirmed by the *p* value of the Jarque–Bera test. A lack of autocorrelation is not rejected only for the DAX index in years 2006–2017 but this is not the case in second subperiod. We observe high increases of kurtosis and skewness parameters.

For both subperiods we computed unconditional Pearson and Spearman correlation coefficients. Both measures give similar results. The strongest relationships are observed for the European indexes CAC, FTSE and DAX. Regardless the subperiods, the last of them exhibit the strongest correlation with DJIA, whereas the dependence between DJIA and WIG indexes is the weakest. In the next subsection we present short overview of selected events which may be sources of contagion.

### Emphasized significant events

**Fig. 1:**
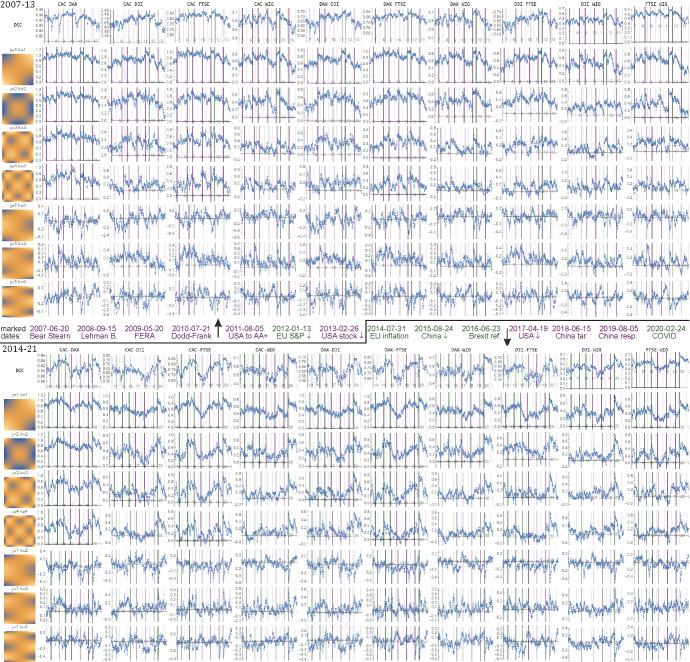
Evolution of correlation coefficients (top: 2007–13, bottom: 2014–21) from the DCC method and some HCR mixed moments (11,22,33,44,12,13,14) for all pairs of indexes, $\eta  = 0.98$ forgetting rate. Left: their corresponding densities $f_{j} \left( x \right)f_{k} \left( y \right)$ (orange—positive, blue—negative). Plots: time evolutions of $a_{{jk}} \left( t \right)$. The $a_{{11}} \left( t \right)$ have similar interpretation as correlation—its evolution is similar to DCC. Further, PCA is used to extract dominating and relatively independent features from them. Grey vertical lines separate the years, green/violet show the marked arbitrarily chosen events. Source: Authors’ calculation

**Fig. 2:**
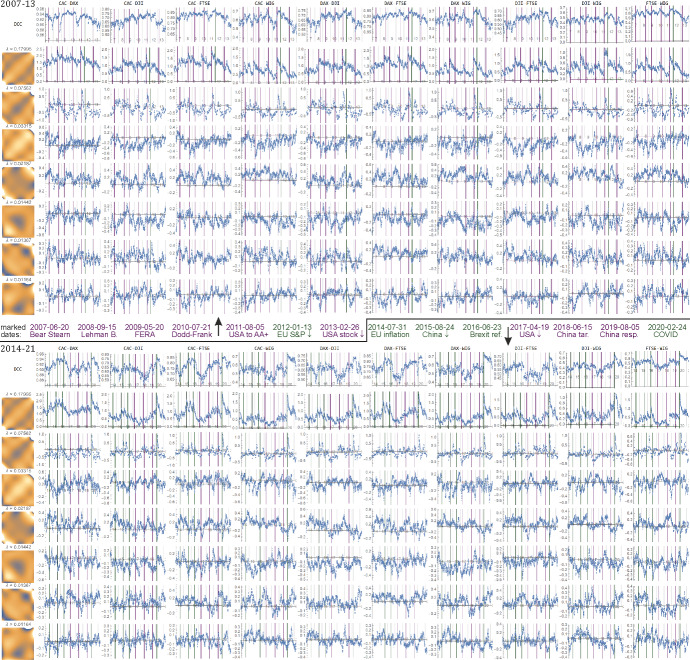
Left: the $\left( {\lambda ,v} \right)$ PCA basis that was found $f_{v} \left( {x,y} \right) = \mathop \sum \nolimits_{{jk}} v_{{jk}} ~f_{j} \left( x \right)f_{k} \left( y \right)$. Plots: time evolutions for all pairs of indexes for DCC and $a_{v} \left( t \right) = \mathop \sum \nolimits_{{jk}} v_{{jk}} ~a_{{jk}} \left( t \right)$ for the first seven eigenvectors $v$. They can be interpreted as dominating and relatively independent features that describe the contagion—various statistical relations between market behaviors. Source: Authors’ calculation

Before moving on to our multi-feature analysis let us choose and discuss some significant events from the periods under investigation in order to be able to appreciate the multi-dimensional description and see that it delivers more detailed information and complements the standard one as DCC. These events are listed in Table 2 and are marked in Figs. 1 and 2.

**Table 2:** Selected important events in the period 2007–2020

Date	Event	Abbreviation
2007-06-20	Bear Stearn bailed out 2 of its hedge funds with $20 billion^a^	Bear Stearn
2008-09-15	Bankruptcy of Lehman Brothers^b^	Lehman B
2009-05-20	President Obama signed the Fraud Enforcement and Recovery Act^c^	FERA
2010-07-21	Dodd–Frank Wall Street Reform and Consumer Protection Act enacted^d^	Dodd–Frank
2011-08-05	S&P downgrade of USA from AAA to AA + ^e^	USA to AA +
2012-01-13	Standard & Poor's downgrades France and eight other eurozone countries^f^	EU S&P ↓
2013-02-26	American stock exchanges evaluated results of Italian elections very negatively^g^	USA stock ↓
2014-07-31	Announcement of bad data about inflation in Euro zone^h^	EU inflation
2015-08-24	Announcements of bad economic data from China^i^	China ↓
2016-06-23	Brexit referendum^j^	Brexit ref
2017-04-19	Announcements of bad economic results of US companies^k^	USA ↓
2018-06-15	Begin U.S.—China Trade War^l^	China tar
2019-08-05	Halting by China purchases of U.S. agricultural products^m^	China resp.
2020-02-24	The coronavirus outbreak spread worsened substantially outside China^n^	COVID

^a^https://www.wsj.com/articles/SB118230204193441422

^b^https://en.wikipedia.org/wiki/Bankruptcy_of_Lehman_Brothers

^c^https://www.lexology.com/library/detail.aspx?g=59adbb21-2922-4eba-9f88-71be5b7e303d

^d^https://en.wikipedia.org/wiki/Dodd%E2%80%93Frank_Wall_Street_Reform_and_Consumer_Protection_Act

^e^https://www.reuters.com/article/us-usa-debt-downgrade/united-states-loses-prized-aaa-credit-rating-from-sp-idUSTRE7746VF20110806

^f^https://www.npr.org/sections/thetwo-way/2012/01/13/145178453/s-p-downgrades-france-deals-a-blow-to-eurozone?t=1618994721386

^g^https://www.brookings.edu/blog/up-front/2013/02/26/italys-election-results-are-bad-news-for-all-of-us/

^h^https://www.marketwatch.com/story/euro-zone-inflation-falls-a-setback-to-ecb-goals-2014-07-31?mod=article_inline

^i^https://www.forbes.com/sites/realspin/2015/08/24/as-stock-markets-crash-from-east-to-west-u-s-and-china-play-the-blame-game/#31d243252f8d

^j^https://www.gov.uk/government/topical-events/eu-referendum

^k^https://countryeconomy.com/stock-exchange/usa?dr=2017-04

^l^https://www.cnbc.com/2018/06/15/trump-administration-to-slap-a-25-percent-tariff-on-50-billion-of-chinese-goods-threatens-more.html

^m^https://www.cnbc.com/2019/08/05/china-reportedly-halts-us-agricultural-imports-in-retaliation-for-trumps-tariff-increase.html

^n^https://www.youtube.com/watch?v=YoDHeMRxidg

We will now give a short descriptive review of the role and impact of the events stated above on the behavior of stock markets in the period under investigation. When creating the sample of events we took into account the predominant role of the US stock exchange and the strenght of the reaction of the prices to the announcements selected. Thus most of these events happened in the USA and almost all of them had a negative impact on prices (indexes) on the day of announcement or the first trading day afterwards. The exceptions were FERA and Dodd-Frank acts which were very important for US stock exchanges. However, their impact on prices was not necessarly negative.

A more detailed description and prediction of the role of these events can be found in Internet sources. Most of these events are expected (based on economic theory) to have a negative impact on stock markets. We will check whether the conatgion takes place and to what extent the results computed using the both methods (traditional and newly developed) coincide or differ in the detection of this phenomenon.

It is important to bear in mind that one of the first events that triggered the subprime crisis of 2007–08 was the collapse of two big hedge funds owned by Bear's Stearns Company Inc. (Jun 20th 2007), which was followed by panic among both individual and institutional investors.

However, the best known and most significant event at the beginning of the GFC was the bankruptcy of Lehman Brothers Bank, whose investments were in housing-related assets and highly leveraged. The day LB (September 15, 2008) announced bankruptcy, DJI closed with the largest single-day drop in many years. This event was followed by a number of liquidity shocks and bankruptcies around the World, especially in the financial sector.

In 2009, President Barack Obama signed several new acts such as FERA (The Fraud Enforcement and Recovery Act of 2009). This had a significant impact on financial markets, not only in the USA but also on other markets. This Act should have protected the financial markets against a repetition of the financial crisis. Some of the rules were restrictive, so the impact of this Act on stock prices was not unique. The indexes declined significantly the next day.

In July 2010 legislation was introduced in the USA based on a proposal of the president Barak Obama known as a proposal for a "sweeping overhaul of the United States financial regulatory system, a transformation on a scale not seen since the reforms that followed the Great Depression". This proposal was supported by Congressman Barney Frank, and by Senator Chris Dodd. Therefore, this legislation is known as the Dodd-Frank Act. This legislation had a significant impact on US financial markets, as well as the prices of stocks traded on this and other markets. The reaction of the markets under investigation was mostly positive at the event and the following day.

On 2011-08-05 S&P downgraded the US federal government rating below AAA for the first time in history. In April S&P had announced a negative outlook on the AAA rating. A few days later its downgrade to AA + was announced. This is not useful for a country because credit becomes more expensive. This decision also had serious consequences for the financial markets. The prices reached a low level not seen in many months. This reaction was extremely noticeable on the Monday—the first trading day after the decision on the Friday.

On January 13th, 2012 nine of the members of the euro area underwent a cut in their ratings by S&P (France, Austria, Malta, Slovakia and Slovenia lost one notch, Italy, Portugal, Spain and Cyprus were downgraded by more than two-notches). S&P was afraid that the measures undertaken by the EU were insufficient to overcome the problems in the euro area. The decision caused a decline in all indexes.

Political events such as elections usually have a significant impact on stock markets. An example was the outcome of the Italian election in February 2013 (the election was won by the populists). It was clear to everybody that after the election a period of serious uncertainty for Italy, for Europe, and also for the U.S should be taken into account. A further election was expected in Italy a few months later. The uncertainty had a negative impact on prices on stock markets in Europe.

Reports about disinflation and deflation in 2014 were a source of special concern for economists. Deflation has a serious impact not only on the real economy but also on financial markets and the propensity of potential investors to be active on financial markets. However, views concerning the impact of deflation on an investor’s activity are not unique. In Tokyo a boom on the financial market was accompanied in different periods by inflation or deflation as well. These reports caused serious declines in all indexes under investigation.

The problems of the stock market in China started on 24 August 2015. The warning of the securities regulator in China, addressed at individuals who played on the stock market for borrowed money, caused declines on the stock market in China. Bad news from China implied a poor performance not only of the Chinese Stock Market but also of international financial markets.

One of the most important political and economic events of the twenty-first century has been Brexit. One could predict stock price changes (declines) on 24 June 2016, the first trading day after the referendum. All indexes declined extremely significantly. Since the 2016 referendum, the FTSE 100 has essentially performed worse than other European indexes. In this time period the German DAX 30 and the French CAC 40 have increased by approximately 40 per cent, while the FTSE 100 only by 20 per cent.

The announcements of the poor performance of companies listed on the stock exchange was one of the most important reasons for the drop in the prices of these companies. The disappointing data concerning US companies announced in April 2017 caused a decline in US indexes. Since the American stock exchanges dominate the world one can suppose an impact of these events on other stock exchanges.

One of the most influential events of 2018 was the U.S—China Trade War. President Trump announced on June 15 that the United States would impose a 25% tariff on $50 billion of Chinese exports. This and the following tariffs hit the global financial markets hard for a whole year. This must be visible in the results obtained by applying novel methodology.

The next important stage of this war took place on August 5, 2019 when China stopped purchasing U.S. agricultural products. In addition, the U.S. Treasury detected for the first time since 1994 that China was manipulating its currency.

Since the end of 2019 more and more events connected with the Covid 19 pandemic have gained importance. On Monday, 24 February 2020 and over the following days the news that the spread of coronavirus had worsened substantially outside China over the weekend made the headlines. The indexes declined rapidly.

The actual reaction of stock markets to respective announcements is not always in line with expectations. We emphasize that the role of these events has been verified in our paper by their impact on correlations/dependence and not in the framework of the event study methodology, such as e.g. in Gurgul and Wójtowicz (2014,2015), Harju and Hussain (2011), Nikkinen et al. (2006).

The main goal of the computations below is to verify the existence of contagion and its strength surrounding the events mentioned using a traditional and a new method described in this paper, and to compare their performance.

## Contagion: empirical results

### Conditional correlations

We apply the model presented in Sect. 3.1, with the time series previously filtered using Vector Autoregressive Models (of order 1). All estimated parameters of the univariate, multivariate models and the shape parameter of the copula are significant. The skewed version of *t* distribution outperforms symmetric distribution, similarly *t* copula is a better choice than the Gaussian copula (according to the information criterion). As a result, we obtain conditional correlations. They are presented in Figs. 1 and 2 together with the proposed features.

The strongest dependence is observed for CAC-DAX with a minimum value of 0.849 and a maximum value of 0.958 for first subperiod and with a minimum value 0.855 and maximum 0.934 for second, the weakest for pair DJI-WIG with a minimum and maximum value equal to 0.207 and 0.602 respectively for first subperiod and 0.363 and 0.634 for second. This is in line with the sample correlation coefficients. The similarity of some plots is also visible, for example CAC-WIG and DAX-WIG or CAC-FTSE and DAX-FTSE. This is the case for both subperiods. Using the Bai and Perron (2003) test one can find changes in the correlation levels. Thus an optimal partition is found (unknown break points of structural changes). Regardless the subperiods, using this procedure we found at least 3 breakpoints (in most cases 4) in the levels and at least 4 (in most cases 5) breakpoints in the trends (to save space these results are available from the authors upon request). All these observations confirm dynamic and very complex dependence patterns.

### Multi-feature analysis

Some channels of contagion based on moments are theoretically possible (e.g. Fry-McKibbin et al. 2019). One can test for contagion by comparing the expected returns of market A to the those of market B. The second channel known as the coskewness channel of contagion takes changes in coskewness into account. They arise from the interaction between expected returns and volatility across markets. Changes in the relation between the returns volatility of one market and the returns volatility of another market can provide an important picture of contagion. After the shock these changes may fluctuate from negative to positive. This is the so-called covolatility channel in the crisis period. These three separate channels of contagion are based on moments. Applying the HCR method we obtain a picture of total contagion by their components based on moments of stock returns.

**Fig. 3:**
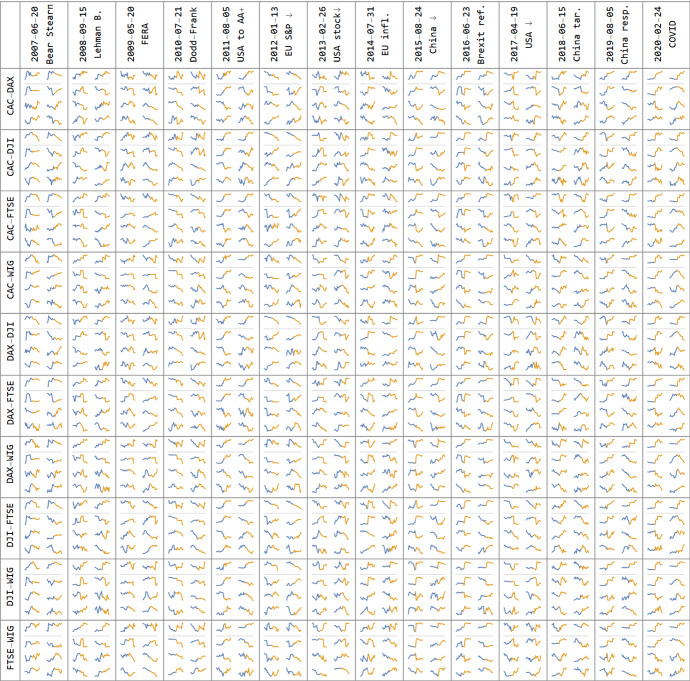
Plots of discussed features for all index pairs around the chosen significant events: 10 days before (blue) and after (orange). Top two are DCC and PCA1, usually having similar behavior. Bottom 6 are PCA2, …, PCA7—dominating and nearly independent statistical features, providing a complementary qualitative description of the reaction of markets. Source: Authors’ calculation

**Fig. 4:**
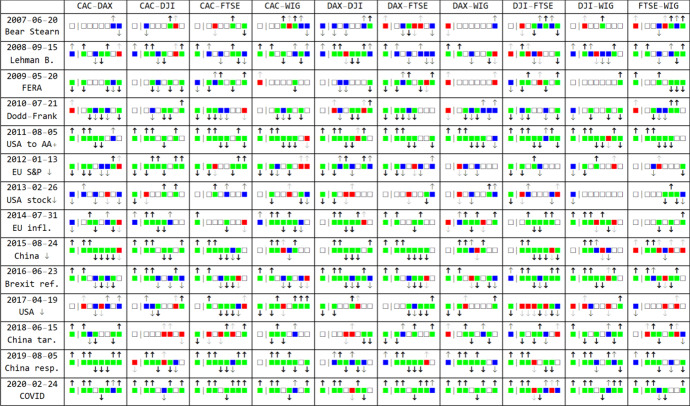
Results of testing indicator changes in the subperiods for 10 pairs of indexes before and after selected significant events in the period 2007–2017. An empty square indicates the hypothesis for 5% significant level is accepted, a red square indicates accepting for 1% but rejecting for 5%, blue square accepting for 0.1% but rejecting for 1%, green square rejecting for 0.1% for DCC, PCA1, …, PCA7 (from left). In cases of a rejected Hypothesis, sign and strength of the change is marked with arrow up or down. As markets are multidimensional systems, these features complement standard DCC by indicating more subtle events in the additional dimensions described. Source: Authors’ calculation

**Fig. 5:**
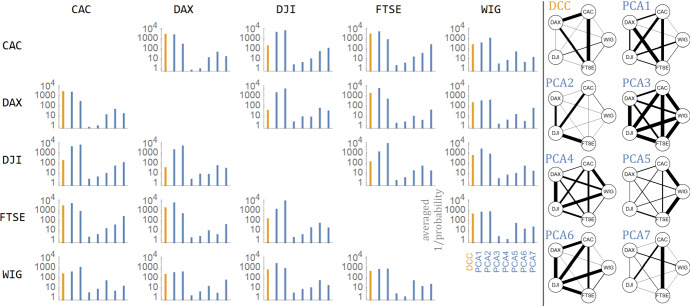
Summary of discussed features and index dependencies based on the 14 selected events. Specifically, using CDF of Student’s t-distribution, there were calculated *p* values for rejection of hypotheses discussed in Fig. 4. The left hand side part of the diagram presents 1 over geometric average over the 14 events of such *p* values for various index pairs and 8 discussed features: DCC (orange) and PCA1 to PCA7 (blue)

In this subsection we present the results of our computations and show them in Figs. 1, 2, 3, 4 and 5. These figures are to a large extent self-contained. However, we comment on them in detail also in the following paragraphs.

We use data normalized with GARCH models. While DCC focused on one type of behavior: evolution of correlation, using HCR instead we can look at the evolution of $a_{{jk}} \left( t \right)$ for various mixed moments, with $j = k = 1$ similar to a standard correlation analysis.

The empirical results concerning the impact of these particular events on stock markets are presented below.

Using higher mixed moments, in theory up to $m = \infty$, especially the higher ones can be just a noise, hence we are focusing on the lower ones up to kurtosis $m = 4$. Figure 1 contains their evolution for some chosen mixed moments.

We can see that many of them have a similar time evolution, hence we then use PCA to extract dominating and relatively independent features. The $25 \times 25~$ covariance matrix $C$ for PCA was found as one common for all—from union of $\left\{ {a_{{jk}} \left( t \right)} \right\}$ over all index pairs. Figure 2 contains the plot for the first 7 such eigenvectors $Cv = \lambda v$, corresponding to the highest eigenvalues $\lambda  > 0$, which are mean variances of the features found $a_{v} \left( t \right)$. Each $a_{v} \left( t \right) = \mathop \sum \nolimits_{{jk}} v_{{jk}} {\text{~}}a_{{jk}} \left( t \right)$ describes the perturbation from uniform joint distribution towards $f_{v} \left( {x,y} \right) = \mathop \sum \nolimits_{{jk}} v_{{jk}} ~f_{j} \left( x \right)f_{k} \left( y \right)$ function from basis. Focusing on this single $a_{v} \left( t \right)$ time evolution, we can imagine joint density as:

9 $$ \rho _{t} \left( {x,y} \right) \approx 1 + a_{v} \left( t \right)~f_{v} \left( {x,y} \right). $$

Hence, the density plots $\rho _{v} \left( {x,y} \right)$ on the left of Fig. 2 provide the intepretations for $~a_{v} \left( t \right)$. Values of variables normalized to nearly uniform distribution on $\left[ {0,1} \right]$ can be thought as quantiles, ½ as median.

This allows at least some of these features to be descibed intuitively through their $f_{v} \left( {x,y} \right)$ densities:

**PCA1**—the first one has $\lambda  \approx 0.18$, which is more than twice larger than the second—this is defnitely dominating behavior, so we can think of it as the **main contagion**. Its $f_{v} \left( {x,y} \right)$ density has increased probability near the diagonal, more localized than $k = j = 1$ correlation-like density in Fig. 1, hence its $a_{v} \left( t \right)$ evolution describes a stronger dependence than correlation—statistical tendency to have a similar quantile for both indexes.

**PCA2** has mainly minimum in top-right corner, and maximum in bottom left, hence it mostly controls imbalance for tail behavior—can be thought of as **imbalance tail contagion**,

**PCA3** also focuses on tails, but in a more symmetric way—a kind of **symmetric tail contagion**,

**PCA7** is interesting as the only anti-symmetric one here: changes sign if switching indexes (the remaning are symmetric), hence it indicates a direction from one index to another; we can think of it as **directional contagion**.

In Fig. 3 shows the magnification of Fig. 2 plots around the significant events listed in the period under study. Their behavior reflects the impact of the events on DCC correlations and PCA1 (top two small plots) for all ten pairs of indexes: CAC, DAX, DJI, FTSE and WIG separately. In addition, for every pair six small plots are given that visualize the behavior of PCA2, …, PCA7—dominating and nearly independent statistical features. As one can see, DCC correlations and PCA1 values are in line to a large extent. Both indicate that most of the events listed in Table 2 are turning points with respect to measures of dependence (correlation), and the values of these measures change after reaching turning points, which can reflect contagion effects. The remaining PCA2, …, PCA7 provide additional complementary information, often reflecting events in various ways.

The results shown in Fig. 3 were tested taking into account the event windows of 5, 10 and 20 days in length around the event. The results for these lengths of windows are similar, so for the sake of brevity we report them for event window 10 (10 day before and 10 days after event day) only. We conducted the same tests for all 8 features (DCC, PCA1, …, PCA7). The null hypothesis states: the average of the given indicators for the 10 days before the event day is the same as the average over the 10 days after the event day. The alternative hypothesis is: the indicators after the event day are on average different than before this day. To visualize the strengths of the effects, in Fig. 4 there 3 significance levels were used: an empty square indicates that the hypothesis for 5% significance level is accepted, colors indicate rejection for growing significance levels: red—weak for 5%, blue—medium for 1% and green—strong for 0.1%. Thus Fig. 4 provides a simple classification for plots from Fig. 3, which also contains additional event indicators like steep slopes or local extrema, all of these could be used for example to build predictors of some objective properties.

As we see the strongest contagion effects reflected in the rise in DCC correlation and rise in PCA1 indicators after the event day can be observed in the case of the collapse of Lehman and Brothers Bank, the downgrading of the ranking of the US economy to AA + and the Brexit referendum (in this case the null for PCA1 is rejected for all pairs of indexes, whereas for DCC only for five). Both indicators increased significantly for all 10 pairs of indexes. This result was supported by a considerable number of significant changes in values of PCA2-PCA7 around the event day for the pairs of indexes under investigation. Relatively strong support for the contagion effect is also noticeable around the time of the publication of bad Chinese economic data on 2015-08-24 and Dodd-Frank Act on 2010-07-21 (reflected in the number of rejections of null for PCA2-PCA7). However, while the Chinese data caused negative performance in the indexes under investigation, Dodd-Frank Act initiated simultaneous, positive performance in these indexes.

Regarding the period 2018–2020 we observe strong support for the contagion effect when China halted US agricultural products on 2019-08-05, and the COVID event on 2020-02-24. For the first of these events we observe a significant increase in correlation for 7 pairs (we fail to reject the null in two cases) while PCA1, PCA 2 increase, whereas PCA3 decreases significantly for all pairs under investigation. In the case of the COVID event the results of testing for DCC and PCA1 are identical. Both indicate a significant increase at a level of 0.1%. This is the case for PCA2 as well. Additionally, we observe a clear pattern for PCA4. In all cases we reject the null and conclude that there was a significant decrease in this feature.

Let us comment on the pictures in Fig. 5. The right-hand side panel in this figure shows a visualization of averaged *p* values (normalized by maximal value) for various index pairs and individual features. The thicker the solid line, the more likely the respective contagion for the events discussed. A graph illustrating contagion by the DCC method indicates that the most (inter)correlated are the largest European indexes. Optimized and related PCA1, describing main contagion, additionally strengthens dependency with DJI, and usually provides a stronger indication of the events discussed. PCA2, which describes imbalance tail contagion, is related to DJI, PCA3 represents symmetric tail contagion. PCA5 is related strongly to WIG, PCA7—the only antisymmetric contagion which indicates direction focuses on CAC. PCA4 and PCA6 are somewhat irregular. These principal components allow different types of contagion to be distinguished and described, which is characteristic of various indexes.

From this figure we can see that PCA1 is probably a better indicator than DCC for detecting this kind of event. In addition, the orthogonal PCA2 and other principal components provide complementary information about contagion and its strength throughout the markets under investigation.

The findings provide significant evidence of contagion through the channels of moments of higher order. The empirical results—in spite of the difference in trading hours between DJI and the European stock exchanges—favor the opinion that the US stock market is probably the most influential and least sensitive to foreign shocks in relation to the stock markets selected in this study. The least stable market and probably the most sensitive to all events seems to be Warsaw Stock Exchange (relatively high probabilities of contagion from all markets under investigation via different channels). The remaining indexes of European developed economies (DAX, CAC, FTSE) are strongly interrelated around shocks (high probabilities in pictures of DCC and PCA1).

To summarize, besides correlation, the effect of contagion on financial markets can be described using multiple mixed moments. As one can see from the empirical results, this process can follow via many channels and can affect not only the rates of returns, but also variances (which represent risk, especially systemic risk) and other descriptive statistics of returns like skewness and kurtosis. The HCR methodology proposed takes most of these complex interdependences into account simultaneously, allowing for a multi-dimensional quantitative description.

## Conclusions

A contagion is usually understood as the spread of an economic crisis from one market or region to another market or region. It can take place on either a domestic or international level. Most investors, economists and analysts understand contagions as being primarily symptomatic of global market interdependence. A contagion is usually associated with financial crises. Contagions can be recognized as negative externalities diffused from one crashing market to another.

In this paper the latter understanding of contagion is the subject of empirical research based on five indexes. Financial markets experience extremely complex hidden dynamics. This suggests that highly parametrized models should be used to try to describe them. While the standard DCC approach describes contagion with the evolution of a single feature (conditional correlation), we have proposed its expansion into multiple features, providing additional information about these hidden multidimensional dynamics. This takes the contagion into account not only with respect to the expected returns but also indirectly via higher moments which determine the distribution functions of the financial variables under investigation in different financial markets.

The channels of propagation of crises across firms, sectors and markets depend on the sources and the nature of the crisis. The new HCR-based method takes the behavior of a number of moments of financial variables chosen by the researcher into account, e.g. returns, and thus allows the complex nature of contagion to be proved with respect not only to mean, but also to risk and volatility simultaneously. From the point of view of visual inspection, the contagion process in its complexity may be understood as adjustments of the distribution curve of a financial variable e.g. returns from one market to the distribution curve of this variable on another market.

Future research should focus on the use of such features for retrospective complex investigations of the differences and similarities between financial and, in general, economic crises accompanied by contagion processes in the past. It can also be extended from pair-wise, to triple-wise and higher order index dependencies. The HCR method offers promising research prospects concerning the impact of the COVID-19 pandemic on the world economy, especially with respect to financial contagion processes on international financial markets (see Dragota and Tilica 2014).

Phenomena on financial markets usually get ahead processes in real economy. The contagion effects on financial markets are important because they can have serious negative consequences for real economy. The channels of contagion, its strength, speed of propagation and impact on real economy are important future research topics.

## Footnotes

1 We estimated many models with different univariate and multivariate distributions. According to the Akaike information criterion we selected skewed t distributions for margins and t-copula to describe the dependence structure. In this way for comparison purposes we use the DCC-copula model that outperforms the classical DCC model of Engle.
